# Overlapping Neural Endophenotypes in Addiction and Obesity

**DOI:** 10.3389/fendo.2017.00127

**Published:** 2017-06-14

**Authors:** Andréanne Michaud, Uku Vainik, Isabel Garcia-Garcia, Alain Dagher

**Affiliations:** ^1^Department of Neurology and Neurosurgery, Montreal Neurological Institute, McGill University, Montreal, QC, Canada; ^2^Faculty of Social Sciences, Institute of Psychology, University of Tartu, Tartu, Estonia

**Keywords:** obesity, addiction, impulsivity, brain, personality and neurocognitive characteristics

## Abstract

Impulsivity refers to a tendency to act rapidly without full consideration of consequences. The trait is thought to result from the interaction between high arousal responses to potential rewards and poor self-control. Studies have suggested that impulsivity confers vulnerability to both addiction and obesity. However, results in this area are unclear, perhaps due to the high phenotypic complexity of addictions and obesity. Focusing on impulsivity, the aim of this review is to tackle the putative overlaps between addiction and obesity in four domains: (1) personality research, (2) neurocognitive tasks, (3) brain imaging, and (4) clinical evidence. We suggest that three impulsivity-related domains are particularly relevant for our understanding of similarities between addiction and obesity: lower self-control (high Disinhibition/low Conscientiousness), reward sensitivity (high Extraversion/Positive Emotionality), and negative affect (high Neuroticism/Negative Emotionality). Neurocognitive studies have shown that obesity and addiction are both associated with increased impulsive decision-making and attention bias in response to drug or food cues, respectively. Mirroring this, obesity and different forms of addiction seem to exhibit similar alterations in functional MRI brain activity in response to reward processing and during self-control tasks. Overall, our review provides an integrative approach to understand those facets of obesity that present similarities to addictive behaviors. In addition, we suggest that therapeutic interventions targeting inhibitory control may represent a promising approach for the prevention and/or treatment of obesity.

## Introduction

**Figure 1:**
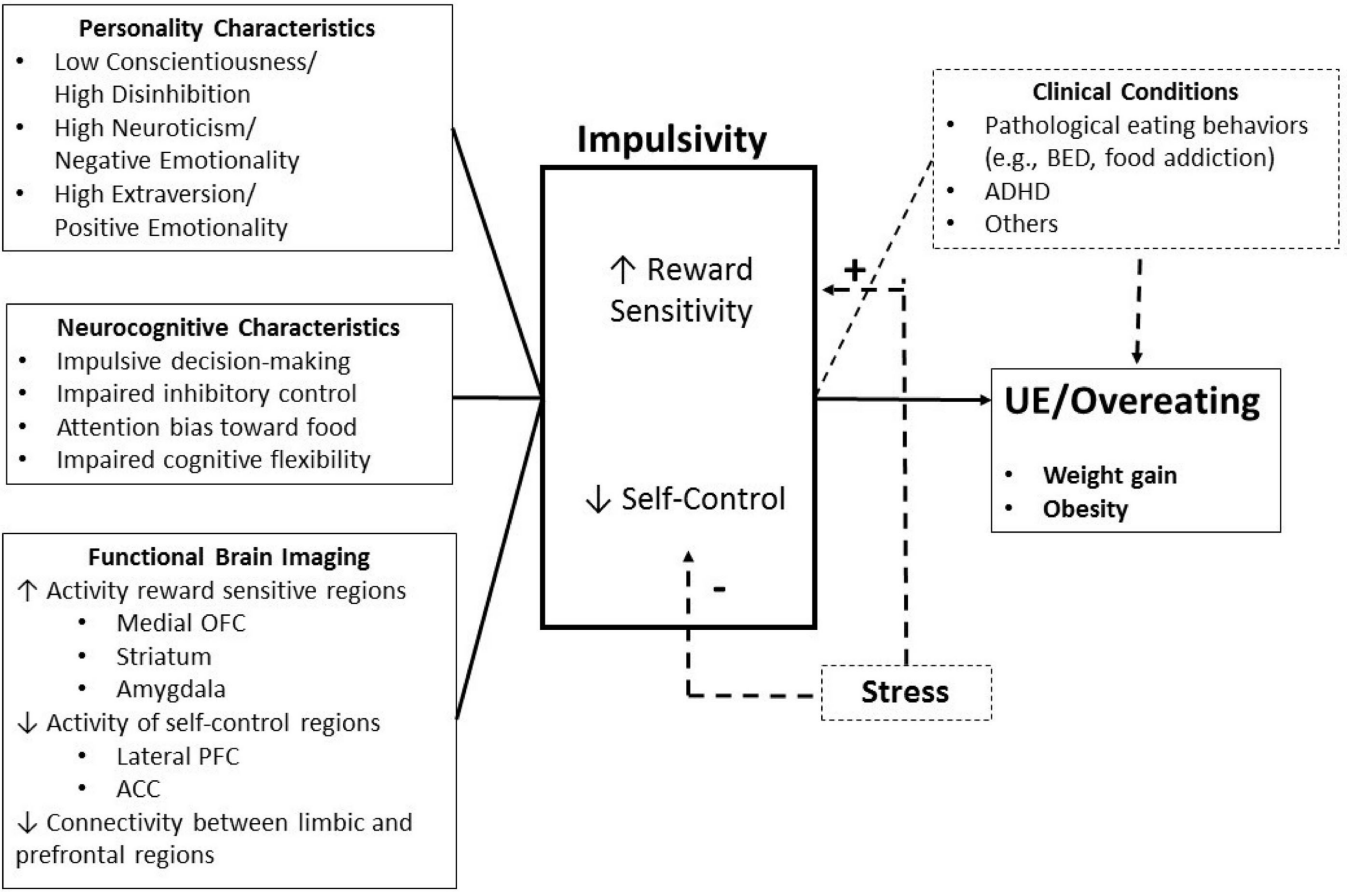
Brain endophenotype of obesity vulnerability. Personality, cognitive, and functional brain imaging characteristics that increase obesity vulnerability. Uncontrolled eating (UE) results from an interaction of elevated reward sensitivity and poor self-control. OFC, orbitofrontal cortex; PFC, prefrontal cortex; ACC, anterior cingulate cortex; BED, binge-eating disorder; ADHD, attention deficit/hyperactivity disorder; BMI, body mass index.

Obesity and addiction are complex and heterogeneous conditions at the intersection of biology and mental health. A bulk of scientific literature has highlighted the importance of neurobiological and neuropsychological factors in the pathophysiology of obesity (Figure 1) (1, 2). More importantly, growing evidence suggests that obesity shares common mechanisms with addiction in terms of neurobiological systems that underlie reward and self-regulation processes (3–5). The goal of this review is to critically assess the putative overlaps between addiction and obesity in four domains: (1) personality research, (2) neurocognitive task, (3) brain imaging, and (4) clinical evidence.

## Brain Mechanisms of Appetite Control and Under Control

Three interconnected brain systems control food intake and eating behavior: (1) the hypothalamus, which responds to internal energy-balance signals, (2) the limbic system [amygdala/hippocampus, insula, orbitofrontal cortex (OFC), and striatum], which is involved in learning and memory and encodes the value or incentive salience of foods, and (3) the cortical (mostly prefrontal) cognitive control system, which enables behavioral self-regulation (6, 7). The normal function of these systems maintains energy homeostasis, enables learning about the nutrient content of foods, and promotes motivation to seek and consume foods as appropriate.

However, individual differences in neurobiological mechanisms involved in the control of food choices and food intake likely explain why some individuals are more susceptible to weight gain than others (8). Indeed, obese individuals may have neurocognitive characteristics that predispose them to overeating upon exposure to favorable environmental or endogenous conditions. One such characteristic is impulsivity. Although many definitions exist (9–14), impulsivity is generally considered as the tendency to act rapidly without full consideration of consequences (15). Sharma et al. (16) recently conducted a meta-analytic principal-components analysis and proposed that impulsivity is a multidimensional construct that includes various distinct psychological components such as disinhibition, neuroticism, extraversion, sensation seeking, inattention, impulsive decision-making, insufficient inhibitory control, and lack of cognitive flexibility (16–19).

Impulsivity is a key component of several neuropsychiatric disorders such as attention deficit/hyperactivity disorder (ADHD), mania, and personality disorders (20, 21). Numerous studies have reported that impulsivity, a personality trait generally observed in individuals with addiction (22–26), may also be associated with high-calorie dietary choices, undercontrolled eating, and the development of obesity (27–31). For instance, individuals characterized by frequent disinhibited behavior and elevated response to potential rewards may be more vulnerable to develop unhealthy weight gain when exposed to the so-called “obesogenic” food-abundant environment (8, 28, 32). Neurobehavioral processes that lead to impulsivity result from the interaction of high arousal response to potential rewards (i.e., reward sensitivity) and poor self-control (i.e., rash spontaneous impulsivity) (14, 28). The reward system is generally thought to encompass projection sites of mesolimbic dopamine neurons, while self-control is dependent on the prefrontal cortex (PFC), especially the lateral PFC, and dorsal anterior cingulate cortex (ACC). Individual differences in impulsivity might constitute a common denominator across obesity and drug addiction. In this regard, several studies have suggested the existence of similarities between addiction and obesity in reward processing (4, 5, 33, 34). In fact, addictive drugs are thought to be addictive by virtue of their actions on neural systems that primarily control appetitive responses to natural rewards such as food (4, 34–36). Dopamine circuitry plays an important role in encoding the reinforcing values of addictive substances (37, 38).

Considering that some neurobehavioral characteristics that confer vulnerability to addiction may also represent risk factors for obesity, this review is aimed at tackling the following question: is the impulsive and poor self-control phenotype identified in drug addiction also present in obesity? The next sections review the evidence in terms of personality, neurocognitive tasks, neuroimaging, and clinical evidence.

## Personality Characteristics

Personality traits reflect tendencies for cognitive, emotional, and behavioral responses to events and environments. Traits that capture impulsive tendencies have been associated with unhealthy weight gain and addiction (39). A recent meta-analytic principal component analysis of personality questionnaires identified three distinct impulsivity subdomains (16): (1) Disinhibition versus Constraint/Conscientiousness, (2) Neuroticism/Negative Emotionality, and (3) Extraversion/Positive Emotionality. These dimensions map well to the “Big Five” personality framework (40), the UPPS (Urgency, Perseverance, Premeditation, Sensation Seeking) scale (19), and many other impulsivity conceptualizations (9, 11). Therefore, we use this three-factor decomposition of impulsivity (16) as a base framework to organize evidence that personality-measured impulsivity is associated with addiction and obesity (Table 1).

**Table 1:** Summary of the main associations between addiction or obesity and impulsivity measurements.

	Addiction	Obesity
**Personality characteristics**		
High Disinhibition and low Constraint or Conscientiousness	+	+
High Neuroticism/Negative Emotionality	+	+, NS
High Extraversion/Positive Emotionality	+	+, NS
**Neurocognitive characteristics**		
Impulsive decision-making	+	+
Impaired inhibition	+, NS	+
Inattention	+	+, NS
Impaired set-shifting	+, NS	+
**Functional brain imaging**		
Reward/motivation system		
Medial OFC/VMPFC	↑ Activity	↑ Activity
Striatum	↑ Activity	↑ Activity
Amygdala	↑ Activity	↑ Activity
Self-regulation system		
Lateral PFC	↓ Activity	↓ Activity ↓ OFC–PCF connectivity
ACC	↓ Activity	↓ Activity
	↓ ACC–striatum connectivity	

*+, positive associations; NS, association not significant; ↑, increase; ↓, decrease; OFC, orbitofrontal cortex; VMPFC, ventromedial prefrontal cortex; PFC, prefrontal cortex; ACC, anterior cingulate cortex*.

### High Disinhibition and Low Constraint/Conscientiousness

The Disinhibition versus Constraint/Conscientiousness factor is comprised of two subfactors associated with behavioral dyscontrol: lack of planning, leading to an inability to refrain from hasty actions, and a lack or perseverance, leading to an inability to maintain self-control in the face of adversity (16). This factor relates to the following measures from commonly used personality scales: lack of perseverance and lack of premeditation from the UPPS, low Conscientiousness from the NEO-Personality Inventory-Revised NEO-PI-R, and motor impulsivity and non-planning impulsivity from the Barratt Impulsiveness Scale (BIS) (16).

Low scores on Conscientiousness have been related to various addictive behaviors (41) including illegal substance abuse (42–44), gambling problems (45), smoking (46–48), and alcohol use (49, 50). Furthermore, lower Conscientiousness increases the risk of relapse after treatment (51). Lack of planning or premeditation assessed using the UPPS scale is also an independent predictor of addiction (52). Thus, the high Disinhibition and low Conscientiousness domain of impulsivity is consistently associated with a higher risk of addiction, supporting the importance of self-control in resisting drug abuse.

Similarly, obesity has consistently been associated with a reduced level of Conscientiousness (28, 53) as measured by the NEO-PI, an association confirmed in a large meta-analysis involving close to 50,000 individuals (54). In a large heterogeneous sample using the BIS, Meule and Blechert (31) found that higher attentional and motor impulsivities were predictive of higher body mass index (BMI) after statistical adjustment for age and sex. However, the effect was small, and non-planning impulsivity was not significantly associated with BMI (31). Finally, studies using the UPPS have also found an association between BMI and lack of perseverance, which is the inability to persist with challenging tasks (55, 56). Furthermore, higher levels of habitual disinhibition, as measured by the Three-Factor Eating Questionnaire, have been associated with body weight gain over time (57). Disinhibition here refers to a tendency to overeat upon exposure to palatable foods or stressful situations, a trait related to consciousness and self-control. In light of these studies, obesity seems to be associated with high Disinhibition and low Conscientiousness. These traits may increase the tendency of an individual to overeat in certain situations and may complicate the maintenance of behaviors associated with body weight reduction in obese individuals (58).

### Neuroticism/Negative Emotionality

The factor Neuroticism/Negative Emotionality reflects a tendency to act rashly in response to negative emotions and to experience cravings when in negative mood states (16). It is reflected in neuroticism in the NEO-PI-R, negative urgency in the UPPS, and attentional impulsivity in the BIS (16).

Neuroticism (NEO-PI-R) has been related to various addiction syndromes, including substance abuse (42–44), problem gambling (45), smoking (46–48), and alcohol use (49, 50), and also with increased risk of relapse after treatment (51). Other studies have also reported an association between negative urgency (UPPS) and substance addiction (59–62). In sum, individuals with addictive behavior may engage in drug use as a way of coping with stress and negative emotion.

The relationship between obesity and neuroticism is less evident. While previous reviews have reported a link between the two (28, 53), a recent meta-analysis found no association (54). A possibility for this lack of significant relationship is that body weight is specifically linked only to some facets of negative emotionality. For example, it has been consistently shown that only the impulsiveness subfactor (“N5:Impulsiveness”) of the NEO-PI-R correlates with adiposity (39, 63). Findings from the UPPS support this notion, as negative urgency, a tendency to experience strong impulses during negative affect, has been linked to greater BMI (55, 56). Other factors that could obscure the link between obesity and Neuroticism/Negative Emotionality include the fact that the association may be present only in women and that neuroticism may also predispose to underweight, *via* a link to eating disorders (64). This could obscure a linear relationship between obesity and neuroticism in population studies. Finally, the link between neuroticism and obesity could be driven by two questions in the Neuroticism scale of the NEO PI-R that specifically target uncontrolled eating (UE) behavior (65, 66).

In summary, the association between the Neuroticism/Negative Emotionality domain and obesity is somewhat less consistent than that with Conscientiousness and Disinhibition. Nonetheless, this personality trait may predispose an individual to overeating in conditions of emotional distress (67), which may lead to adiposity in the long term.

### Extraversion/Positive Emotionality

The Extraversion/Positive Emotionality factor refers to sensation seeking and sensitivity to appetitive or rewarding cues (16). Individuals with high Extraversion/Positive Emotionality are sensitive to positive environmental stimuli and more likely to engage in impulsive or reward-seeking behaviors when they experience positive emotions. They are said to seek novel and exciting experiences. Extraversion/Positive Emotionality correlates with the Extraversion domain in the Five-Factor Model of personality and with Sensation Seeking of the UPPS (16). The Sensitivity to Reward portion of the Sensitivity to Punishment and Sensitivity to Reward Questionnaire (SPSR) is a self-report questionnaire that also assesses this dimension (28, 68).

Numerous studies suggest that reward-driven impulsivity represents a risk factor for both drug addiction and overeating by enhancing the motivation to obtain drugs or palatable foods (69, 70). Higher scores in Extraversion have been related to drug addiction (47). A related trait, positive urgency, the tendency to act rapidly in response to positive emotions, was also correlated to substance addiction (59–62). In addition, Sensation Seeking is commonly associated with substance-use disorders and alcohol problems (62). In sum, the literature is consistent in associating the Extraversion/Positive Emotionality domain of impulsivity to addictive disorders.

Some studies have proposed that high BMI is associated with increased levels of Extraversion (28, 53). Higher scores in Extraversion also seem to predict prospective weight gain (after 2 years) (71). However, contradictory findings do exist, with a meta-analysis (54) failing to show a consistent relationship between obesity and Extraversion in longitudinal studies. However, Davis et al. (72) found that reward sensitivity, as assessed by the SPSR, was associated with maladaptive eating behaviors such as preference for high-calorie foods and overeating (72). They suggested that some individuals may have greater reactivity to food cues and that weight management, in these individuals, may represent a continuous struggle in the modern obesity-promoting food environment. Using the SPSR, this group also demonstrated an inverted U-shaped relationship between reward sensitivity and BMI in a sample of subjects covering a large spectrum of adiposity values, suggesting that lean and severely obese subjects were less sensitive to reward than overweight and obese subjects (73). By using the Behavioral Activation Scale, other groups have also provided evidence of a quadratic relationship between BMI and reward sensitivity (74, 75). To explain this curvilinear relationship, Davis and Fox (73) proposed that both hyper- and hyposensitivity to reward could predispose to obesity. The possibility of an inverted U-shape relationship between BMI and Extraversion suggests that differences in the range of sampled BMI across studies might account for the discrepancies in the literature. In addition to this, gender might modulate the correlation between Extraversion and BMI. For women, lower scores in Extraversion seem to relate to higher adiposity (76, 77), while the opposite has been reported in males (76, 78).

**Figure 2:**
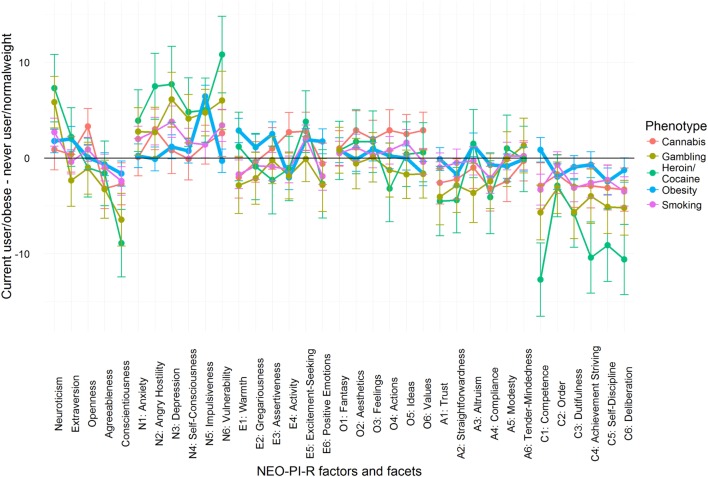
Personality profiles of obesity and addictive phenotypes according to NEO-personality inventory revised. We present the difference in T-score units between obese minus normal weight group and addiction phenotype group minus control group. On a broad factor level, all phenotypes share higher Neuroticism (high Negative Emotionality) and lower Agreeableness and Conscientiousness (high Disinhibition). However, on a finer facet level, the profiles become less similar. For instance, obesity sets apart from other addictions only peaking at one facet of Neuroticism, and not on all facets of Conscientiousness. Therefore, while there are broad similarities, obesity and addictive phenotypes are not fully similar to each other. Mean scores were obtained from these papers (39, 42, 79).

Overall, although contradictory findings do exist, the current evidence points in the direction of similar impulsivity profiles in obesity and addictive disorders. Specifically, these two disorders seem to share lower cognitive control (high Disinhibition/low Conscientiousness), and a tendency toward making impulsive decisions in response to positive (high Extraversion/Positive Emotionality) and negative (high Neuroticism/Negative Emotionality) mood states. Figure 2 displays a comprehensive overview of personality differences in obesity and addiction as derived from Ref. (39, 42, 79). This shows that while, on a broad level, obesity seems to be similar to addictive behaviors, there are also differences at the finer level of personality subscales.

## Neurocognitive Tasks

Laboratory-based neurocognitive tasks can be used to measure inhibitory control or self-regulation. Commonly used examples are the delay discounting task, the stop-signal task (SST), the Go/No-Go task, the Stroop task, and the Wisconsin card sorting task (WCST) (80). These neurocognitive tests assess various dissociable dimensions of impulsivity, including impulsive choice, impulsive responding, and inattention (15, 81). Sharma et al. (16) also performed a meta-analytic principal-components factor analysis of the most commonly used behavioral task measures of impulsivity and they identified four major domains: (1) impulsive decision-making, (2) inattention, (3) inhibition, and (4) shifting. The next sections describe how these four domains of impulsivity are associated with addiction and obesity (Table 1).

### Impulsive Decision-Making

Impulsive decision-making (or impulsive choice) refers to a tendency not to delay gratification and to prefer immediately available rewards (16). It is typically tested with the delay discounting task, in which participants must choose between an immediate, smaller monetary sum and a larger, delayed amount (82). A steeper delay discounting rate is associated with a greater preference for immediate rewards, which reflects impulsive decision-making.

Kirby and Petry (83) have demonstrated using a questionnaire version of this task that substance-addicted individuals have higher discounting rates for delayed rewards than controls. Two meta-analyses also provided strong evidence that steeper impulsive discounting rate is associated with the severity and the frequency of addictive behaviors (84, 85). The magnitude of the association was similar between various types of addictive problems (alcohol, gambling, tobacco, cannabis, opiates, and stimulants) (85). The same group also reported a similar relationship in obesity: although results vary, their meta-analysis concluded that obesity is associated with steeper delay discounting of future monetary and food rewards (86). Interestingly, Weygandt et al. (87) recently found that less functional MRI (fMRI) activation of inhibitory-control areas during a delay discounting task is associated with poor weight loss maintenance in the long term. More specifically, obese subjects seem to have greater delay discounting for food compared to other type of rewards. Similarly, substance-addicted subjects have greater delay discounting for drugs compared to other type of rewards (28, 85, 86). Impulsive decision-making in addiction and obesity may explain why some individuals engage in maladaptive behaviors that are immediately rewarding but detrimental in the long run.

Another perspective in impulsive decision-making revolves around the concept of risk sensitivity. Risk sensitivity refers to the individual degree of attraction or aversion to uncertain outcomes (88). A moderate risk-seeking behavior may confer advantages in the discovery of new environments and resources and might lead to experiencing exciting adventures. However, an excessive attraction toward risk may also be associated with adverse consequences and might have a role in the development of drug addiction. In recent years, the concept of risk sensitivity has been used to describe impulsive behavior in addiction and obesity (89, 90). Both addiction and obesity might involve to some extent an approach tendency toward short-term pleasure despite the risk of long-term negative consequences (89, 91). Several studies have suggested the existence of addiction-related alterations in risky choices. For example, compared with healthy controls, participants who binge drink exhibited increased risk-seeking when anticipating large unlikely monetary losses (92). Risky decision-making and higher delay discounting also appear to hamper the maintenance of abstinence following treatment (93).

Relatively few studies have directly examined risk-taking similarities or differences between addiction and obesity to date. One study found that obese individuals with and without binge-eating disorder (BED) made as many risky choices in a monetary task as drug addicts (94).

### Inhibition

The inhibition domain refers to the ability to suppress prepotent motor responses (16). Tasks that test inhibition include the Go/No-Go and the SST (80, 82). In the Go/No-Go task, individuals are asked to answer as quickly as possible when a repeated visual stimulus appears (Go signal) but to inhibit their response when a rare stop signal appears (No-Go signal). In the SST task, the stop signal is presented after the Go signal to measure the ability of an individual to stop an already initiated response (95).

Considerable evidence links drug addiction to impaired inhibitory control (96–98). A meta-analysis of 97 studies using the SST or Go/No-Go tasks reported that impaired inhibitory control is generally observed in subjects with heavy substance-use disorders and pathological gambling (99). However, there was lack of evidence for inhibitory deficit in subjects diagnosed with cannabis, opioid, or Internet addiction (99).

Similarly, obesity has been linked to poor inhibitory control. A comprehensive literature review found that obese and overweight individuals have lower inhibitory-control performance in food-specific versions of the SST (100). The authors proposed that the SST may be a good marker to identify individuals at high risk of weight gain or less responsive to weight loss interventions (100). Poor inhibitory control is also associated with higher prospective weight gain (101, 102) and food intake (103). Furthermore, a recent meta-analysis confirmed that obese adults display inhibitory-control deficits compared to lean controls (104). Similar findings have been reported in children and adolescents (104–108). However, Loeber et al. (109) found no significant differences between lean and obese participants in performance during a food-related Go/No-Go task. Furthermore, others did not find an effect of BMI *per se* on SST performance in response to food, but rather a complex interaction between BMI and impulsivity (110).

Furthermore, Voon et al. (111) used a serial reaction time task adapted from rodent experiments to assess a somewhat different form of motor impulsivity: waiting impulsivity or premature responding. They found that premature responses were significantly higher in addicted individuals (alcohol, smoking, and drugs) but not in obese or BED subjects. Thus, certain forms of motor impulsivity seen in addiction are not present in obesity.

### Inattention

The third impulsivity domain considered here refers to the ability to focus attention on specific activities while suppressing the response to distracting stimuli (16). The Stroop task is typically used to measure the inattention domain of impulsivity (16). This task requires participants to identify (usually verbally) the color of a written color word, without reading the word itself. When the word is printed in a color that is incongruent with the word (for example, the word blue printed in green), there is a conflict between word reading and color naming. PFC has been implicated in the performance of the Stroop task (112).

A refinement of this task, the “addiction-Stroop,” in which the distractor stimuli represent the addictive substance of interest, has also been used to assess altered attentional processes associated with addictive behaviors (113). Indeed, there is considerable evidence that individuals with addiction have an attentional bias toward drug-related cues, which may play an important role in drug craving, consumption, and relapse (114). Similarly, some studies have reported that obese individuals may have attentional biases toward food-related cues, which may increase food consumption and weight gain over time (115). Hall et al. (116) found that elevated levels of inattention were predictors of high-calorie snack consumption. Furthermore, a recent study demonstrated that obese individuals are characterized by lower scores on the traditional Stroop task (117). Even though some reviews reported inconsistent associations between attentional bias for food-related cues and obesity (28, 115, 118, 119), we previously concluded in a comprehensive review that the Stroop task seems to be one of the most consistent cognitive control tasks demonstrating replicated associations with obesity and weight-related eating behaviors (28).

### Shifting

Behavioral flexibility, or the ability to switch attentional or task set in response to changing rules, has also been linked to impulsivity (16). It is typically evaluated with the WCST (16). During this task, participants are asked to match a response card to one of four category cards based on specific rules (e.g., color, shape, or number) (120). The rules change over time and subjects need to modify their response accordingly. A tendency to fail to switch is called perseveration, and it may reflect a form of impulsivity. Poor cognitive flexibility has been associated with compulsive behaviors (121, 122).

A recent review by Morris and Voon (122) argued that the links between cognitive flexibility assessed using the WCST and addiction are inconsistent. Indeed, some studies reported impaired cognitive flexibility in substance-addicted (123) and non-substance-addicted (gambling, bulimia) individuals (124). However, others found no significant association between performance on the WCST and addiction (125–127). With respect to obesity, a recent study reported impaired performance on the WCST in obese individuals compared to individuals with other eating disorders (128). In addition, a meta-analysis (121) and systematic review (118) both reported impaired WCST performance in obese individuals compared to controls. However, overweight rather than obese individuals were not characterized by set-shifting impairment (121).

Overall, current evidence from neurocognitive tasks is that obese and addicted individuals are both generally characterized by higher impulsive decision-making and attentional bias in response to drug or food cues. In addition, obesity is usually associated with altered cognitive flexibility (set-shifting) assessed with the WCST and poor inhibitory control assessed with the SST.

## Neuroimaging

Neuroimaging has been used to investigate functional and anatomical neural correlates of the vulnerability to drug abuse and overeating. Vulnerability to addiction can be considered as resulting from the interaction of increased incentive response to drug cues, propensity for habit formation, poor self-control, and heightened negative emotionality (129, 130). These processes are related to different but interconnected brain systems: (1) the mesolimbic dopamine system, implicated in reward, motivation, and habit formation, which includes the ventral tegmental area, ventral striatum, anterior insula, OFC, amygdala, and hippocampus and (2) cognitive control circuits, implicated in self-regulation, including middle and inferior lateral PFC, ACC, and insula (131). Previous neuroimaging studies have shed light on the role of the mesolimbic system in the pathophysiology of addiction (132–139). Participants with addiction seem to exhibit increased fMRI activation in ventral striatum, amygdala, and medial regions of OFC in response to drug cues (133). In general, these results are consistent with the observation that participants with drug addictions exhibit a heightened attentional or motivational focus toward drug-related stimuli (130).

With regards to cognitive control circuits, adolescents who initiate substance use seem to exhibit reduced blood oxygen level dependent (BOLD) activity in the dorsolateral prefrontal cortex (DLPFC), putamen, and inferior parietal cortex during a Go/No-Go task, suggesting that baseline dysfunction in these areas could predict the initiation of drug use (140, 141). In this vein, theoretical work has highlighted the key role of PFC areas in the endophenotype of addiction vulnerability (112). For instance, participants with addiction seem to exhibit prefrontal dysfunction, implicating the dorsal PFC (dACC and DLPFC) involved in self-control, the ventromedial prefrontal cortex (VMPFC) involved in emotional regulation and salience attribution, as well as the ventrolateral prefrontal cortex and lateral OFC involved in inhibitory or automatic responses (112). It has been proposed that the PFC is involved in addictive behaviors through its capacity to regulate subcortical regions implicated in reward processes (112, 142). For example, the strength of the connectivity between dACC and striatum has been negatively associated with the severity of nicotine addiction (143). PFC dysfunction might be implicated in an endophenotype named *impaired response inhibition and salience attribution* (112). This endophenotype both increases sensitivity to drug cues and reduces the capacity to inhibit maladaptive behaviors (144). Consistent with these findings, drug craving seems to involve the amygdala, ACC, OFC, and DLPFC (145), suggesting the involvement of both reward-related and inhibitory-control resources.

Numerous brain imaging studies also support the notion that vulnerability to weight gain and overeating may result from the interaction between elevated food reward sensitivity (incentive salience of the cue) and poor inhibitory control. In response to visual food stimuli, participants with obesity exhibit increased activation in the dorsomedial PFC, the ventral striatum, the parahippocampal gyrus, the precentral gyrus, the superior/inferior frontal gyrus (IFG), and the ACC relative to lean subjects (119–121). These brain regions are thought to encode reward responses, incentive salience, motor coordination, and memory. Longitudinal study designs have shown that increased BOLD activity in reward-related areas (i.e., ventral striatum and OFC) predicts weight gain, suggesting a link between heightened reward responsivity and the development of obesity (146, 147). With regards to inhibitory-control circuits, participants with obesity seem to show consistent blunted activity in the DLPFC and insula in response to visual food cues (148), suggesting a reduced engagement of neural resources associated with inhibition, executive control, and interoceptive awareness. Of note, longitudinal studies have reported that increased activation in the DLPFC in response to high-calorie food images is associated with successful voluntary weight loss (149, 150). An interesting possibility is that self-control processes in the DLPFC may downregulate the activity of the VMPFC and thus, modulate eating choices (151). Supporting this model, stronger functional coupling between the DLPFC and the VMPFC has been associated with successful dietary weight loss (102) and healthier dietary decisions (151). Furthermore, other fMRI studies have reported that the regulation of food craving was associated with increased activity in the DLPFC, IFG, and dorsal ACC (152–154).

A few neuroimaging studies in obesity have specifically addressed cognitive control processes by using cued inhibitory-control paradigms. Here, fMRI studies have found negative associations between brain activation in executive-control regions (lateral PFC) and BMI (155–157). Longitudinal studies have reported that activity in the DLPFC during cognitive control tasks seems to predict successful weight loss after treatment (87, 102). Conversely, impairment of cognitive control over appetitive regions may (1) decrease the acquisition of behaviors leading to successful weight loss and (2) enhance the motivation to consume palatable foods, even in the absence of energy requirements (6, 158).

Together, the aforementioned studies suggest that participants with obesity and patients with addictions present similar functional alterations in frontal regions and in mesocorticolimbic circuits. However, to date few neuroimaging studies have directly compared the impact of obesity and various types of addictions on brain activation. This last point is especially relevant, since food and drug cues seem to activate similar brain regions involved in reward processes, such as striatum, amygdala, OFC, and insula (135). A previous meta-analysis observed that participants with obesity and subjects with different forms of substance addiction exhibited similar heightened BOLD activity in the amygdala and ventral striatum in response to the relevant cues (food in obesity and drugs in addiction) (159).

Overall, current fMRI studies provide evidence for the existence of shared neural mechanisms associated with obesity and different forms of addiction. Poor inhibitory control in combination with increased reward sensitivity and attention to cues (foods or drugs) may be relevant for both obesity and addictive disorders.

## Clinical Evidence

### Binge-Eating Disorder

Binge-eating disorder (BED) is an eating disorder characterized by recurrent episodes of consumption of larger than normal amounts of food in short periods of time (160). These binges are associated with a sense of loss of control and subsequent distress and culpability. Many studies report that individuals with BED display increased impulsivity, altered reward sensitivity, and altered attentional and memory biases to food-related stimuli (161, 162). For example, individuals with BED have steeper delay discounting of rewards (163) and lower activation in the PFC regions during inhibitory-control tasks (164, 165), suggesting that impulsivity may be importantly related to BED. BED presents phenotypic similarities with substance-use disorders (166). Indeed, substance-use disorders and BED are both characterized by loss of control over consumption, and chronic overconsumption despite negative consequences (167).

The observation that BED shares behavioral and neural underpinnings with substance-use disorders has led to the use of the expression “food addiction,” specifically with respect to individuals who meet BED diagnostic criteria, but also more generally as an explanation for obesity. The model hypothesizes that hyper-palatable foods may lead to an addictive response in vulnerable and high-risk individuals (168, 169). Individual variations in “food addiction” can be operationalized by means of scales such as the Yale Food Addiction Scale (YFAS) (166, 170, 171) or the YFAS 2.0 (a revised version adapted for the DSM-5 criteria for substance-related and addictive disorders) (172). However, the model of “food addiction” in humans remains controversial (173–177). The main criticism is that the model is based mostly on animal studies and that the type and quantity of food that characterize “food addiction” are imprecise (173, 174, 177). Furthermore, animals rarely exhibit addition-like behaviors toward sugar; these behaviors only occur when access to sugar is intermittent, and not because of some neurochemical effect of sugar (177). This failure in characterizing what constitutes an addictive agent in foods has led to some theorists to advocate in favor of referring to the phenomenon as “eating addiction” instead (178). We have proposed the term “UE” (65). In addition, even though “food addiction” scores are positively correlated with several measures of adiposity (179), not all individuals with obesity or BED exhibit “food addiction,” and conversely, some individuals displaying “food addiction” are not obese (174, 180). Davis (171) suggests that “food addiction” constitutes the last stage of an overeating spectrum (65) and may represent an extreme subtype of BED. In a similar vein, BED has been strongly associated with obesity; however, BED can also occur in individuals with a wide spectrum of body weight (181). As suggested by previous studies, obese individuals with BED seem to represent a specific and possibly rare subtype of obesity (166, 182). Nonetheless, while the lines between BED, “food addiction,” and obesity are ill-defined, these conditions seem to share common characteristics including impulsivity and reward dysfunction.

### Attention Deficit/Hyperactivity Disorder

Attention deficit/hyperactivity disorder is a neurodevelopmental disorder characterized by inattention, hyperactivity, and impulsivity (160). Neuroimaging studies have suggested a link between ADHD and dysfunction in frontostriatal circuits. For instance, anatomical studies have observed that participants with ADHD exhibit cortical thinning in the PFC, associated with inhibitory-control deficits (183, 184). A frequent comorbidity of ADHD is substance-use disorders (185–187). For example, a longitudinal study found that children and adolescents with ADHD are at higher risk of substance-use disorders and tobacco smoking after a 10-year follow-up period (188).

There is also growing evidence of a link between ADHD and obesity. However, this relationship remains controversial (189, 190). A recent meta-analytic report found a significant association between obesity and ADHD in both children and adults after controlling for possible confounding factors (e.g., gender, study design, country, and study quality) (190). Conversely, another recent meta-analysis reported that the strength of the association between ADHD and obesity was weak. Nevertheless, the effect size increases with age suggesting that the association is stronger in adults than children (189). Two longitudinal studies found that individuals with ADHD are at higher risk of obesity than controls (191, 192). A recent systematic review found that the strength of the association between ADHD and disordered-eating behavior was moderate (193). Furthermore, genetic correlations were found between ADHD, BMI, and smoking (194). To explain the link between ADHD and obesity, researchers have hypothesized that these two disorders exhibit common neurocognitive features, such as impulsivity and inattention (195). Davis et al. (196) also suggested that individuals with ADHD may be more inattentive to their internal signals of hunger and satiety, which may lead to subsequent overeating. Interestingly, the pharmacological treatment of ADHD with dopaminomimetics may facilitate weight control by modulating satiety signals and eating behaviors (197). Overall, ADHD appears to be associated with both addiction and obesity and with the neural endophenotypes that predispose to both, namely, self-control deficits and impulsivity.

### Stress or Emotion Dysregulation

Stress is a ubiquitous risk factor across several psychiatric disorders, and it has important implications for our current understanding of addiction and obesity (198, 199). Studies have shown associations between stress and drug craving (200, 201). Chronic exposure to life stressors also predisposes to the transition from casual drug use to substance abuse (202), and it seems to increase the risk of relapse among abstinent users (202). Stress is one of the central elements of the model of addiction proposed by Koob and Le Moal (203). According to this framework, addiction can be conceived as a continuous process of hedonic and homeostasis dysregulation (204). The *spiraling distress* cycle describes how continued drug use along with failures in self-regulation can cause chronic dysregulation of the reward system. As the drug use escalates, patients reach a pathological state that is characterized by increased negative affect and distress, which are particularly pronounced after drug withdrawal. The model hypothesizes that this aversive emotional state constitutes a powerful motivator for drug-seeking, since patients at severe stages of drug addiction will consume drugs to find relief from distress (203).

With regards to obesity, mounting evidence suggests that stress can modify eating patterns (198, 205). Negative mood states or chronic stress increase subjective appetite or food cravings, selective attention toward food, and individual preferences for high-calorie snacks (e.g., sweets and chocolate) (206–209). Increments in food seeking and food consumption during emotionally demanding situations might relate to the fact that eating a so-called “comfort food” promotes improvements in negative affect (210, 211), in line with the model of Koob and Le Moal. The relationship between stress and food intake, however, presents remarkable interindividual variations. Indeed, stress can be associated with both augmented and diminished appetite (205), with around 30% of the population experiencing increases in appetite, 48% appetite suppression, and the rest no change (212). Studies have suggested that obesity constitutes a crucial predictor of increases in food intake during stress. For instance, while work stress has been associated with weight gain in male participants with elevated BMI, the same psychological stressor leads to weight loss in lean participants (213). Finally, individuals with obesity seem to suffer a higher numbers of adverse life events and chronic stressors compared to lean individuals (198).

Stress acts on brain areas involved in both sides of appetite regulation: the reward/motivation system and the inhibitory-control pathways. For example, Tryon et al. (214) found that in response to high-calorie food pictures, women characterized by higher chronic stress have increased activation in brain regions involved in reward and motivation as well as reduced activation in prefrontal regions. These women also demonstrated greater consumption of high-calorie foods after the scanning session. In a similar vein, Maier et al. (215) compared the neural responses between participants assigned to a laboratory stressor versus those assigned to a neutral condition during a food choice task. Subjects assigned to the stressor put greater value on the taste of the food items presented. Paralleling this, bilateral amygdala and right nucleus accumbens reflected the relative taste value of chosen options more strongly in stressed compared to control participants. The authors interpreted these findings as suggesting that acute stress may increase the rewarding attributes of food stimuli (215). Furthermore, Jastreboff et al. (216) observed that obese individuals exhibit increased activation in striatal, insular, and hypothalamic regions in response to stress and favorite-palatable food cues compared to lean individuals. These increased corticolimbic-striatal activations in response to food cues and stress were also positively associated with food craving ratings, suggesting that some individuals may be at higher risk to consume high-calorie foods during stressful periods (216). On the basis of the theoretical model proposed by Sinha and Jastreboff (198), highly palatable food cues in combination with chronic stress exposure could modulate emotions, metabolic responses (e.g., glucose and energy-balance hormones), and stress-responsive hormones (e.g., adrenocorticotrophin cortisol) that influence brain regions involved in reward, motivation, self-control, and decision-making. Thus, stress sensitivity likely interacts with reward systems to promote either drug use or overeating (or both) in vulnerable individuals (217).

## Conclusion

### Evidence of Non-Overlap

Despite the similarities exposed here there is also evidence that obesity and other addictive behaviors differ and may only overlap partially (218). While some studies have observed higher rates of addictive disorders in obese populations (219, 220), others have reported a lack of significant relationships between addiction and obesity (221–224). Methodological aspects (224) as well as the remarkable intrinsic complexity and heterogeneity associated with obesity and addiction (225) might help to explain the discrepancies observed between studies. Multiple factors (e.g., impulsivity and depressive symptoms) might interact with obesity/eating behavior in complex ways that are difficult to account for in studies with relatively small sample sizes. These factors may explain conflicting studies in the literature. Furthermore, an interesting possibility is that some subtypes of obesity might be at higher risk for developing addictive behavior (33). For instance, some post-bariatric surgery patients seem to exhibit increased rates of addictive problems (226–228). This phenomenon is commonly referred to as “cross addiction” or “addiction transfer.”

Limitations of the present review should be acknowledged. Obesity results from a chronic positive imbalance between energy intake and energy expenditure. Almost all studies in obesity and impulsivity presented here describe obese participants in terms of the BMI (kg/m^2^). While the BMI is an indicator of total adiposity, an important disadvantage is that it might not necessarily be associated with addictive-like eating patterns. In this vein, it is thus crucial to include a description of the participants in terms of their eating behavior or their UE patterns. Furthermore, clinical conditions that often present in comorbidity with obesity, such as BED or ADHD are not systematically evaluated and excluded in all the studies included in the present review. This point constitutes an important limitation that might obscure or inflate the overlap between addiction and obesity.

### Concluding Sentences

Addiction and obesity are health problems with high phenotypic complexity. Growing evidence from personality, cognitive neuroscience, and brain imaging studies suggest that the combination of reduced cognitive control and, to a lesser extent, increased reward sensitivity is a risk factor for the development and maintenance of both syndromes. This is especially true in the domain of cognitive control (Figure 2) as measured by the Conscientiousness versus Disinhibition factors on personality questionnaires, by cognitive tasks of executive function, or by diminished recruitment of areas associated with cognitive control, such as the lateral PFC, in fMRI studies. Individuals characterized by high food drive and high cognitive control might better control their body weight in an environment rich in palatable foods.

The present review provides a comprehensive view of impulsivity-related alterations in obesity and addiction, covering results from the personality, neurocognitive, neuroimaging, and clinical fields. The conclusions of the review have the potential to inform clinical approaches aimed at the prevention or treatment of obesity. Diminished self-control is a predictor of poorer treatment outcomes in substance abuse disorders (51) and might also be one in obesity treatment. The findings of the present review might, as such, be of interest to cognitive behavioral therapists aiming to foster impulse control strategies in participants with obesity. Specific inhibitory-control interventions may also represent a promising approach for the prevention of obesity in individuals with poor self-control and high reward sensitivity.

## Author Contributions

AM: design and conception of the manuscript; wrote the manuscript; and gave final approval. UV and IG: wrote and critically revised the manuscript; gave final approval. AD: design and conception of the manuscript; wrote and critically revised the manuscript; study supervision and responsible for funding; and gave final approval.

## Conflict of Interest Statement

The authors declare that the research was conducted in the absence of any commercial or financial relationships that could be construed as a potential conflict of interest.
